# Astodrimer sodium nasal spray forms a barrier to SARS-CoV-2 in vitro and preserves normal mucociliary function in human nasal epithelium

**DOI:** 10.1038/s41598-024-72262-w

**Published:** 2024-09-11

**Authors:** Jeremy R. A. Paull, Carolyn A. Luscombe, Aynaz Seta, Graham P. Heery, Michael D. Bobardt, Philippe A. Gallay, Samuel Constant, Alex Castellarnau

**Affiliations:** 1 Starpharma Pty Ltd, Abbotsford, VIC 3067 Australia; 2 Department of Immunology and Microbiology, The Scripps Research Institute, La Jolla, CA 92307 USA; 3 Epithelix Sàrl, Plan-les-Ouates, 1228 Geneva, Switzerland

**Keywords:** Viral infection, Preclinical research

## Abstract

COVID-19 remains a severe condition for many including immunocompromised individuals. There remains a need for effective measures against this and other respiratory infections, which transmit via virus-laden droplets that reach the nasal or oral mucosae. Nasal sprays offer potential protection against viruses. Such formulations should preserve normal nasal mucociliary function. The antiviral barrier efficacy and effects on mucociliary function of astodrimer sodium nasal spray (AS-NS) were evaluated and compared with other available nasal sprays—low pH hydroxypropyl methylcellulose (HPMC-NS), iota-carrageenan (Carr-NS), nitric oxide (NO-NS), and povidone iodine (PI-NS). Assays simulated clinical conditions. Antiviral barrier function and cell viability were assessed in airway cell monolayers, while a model of fully differentiated human nasal epithelium (MucilAir™) was utilized to evaluate tissue integrity, cytotoxicity, cilia beating frequency, and mucociliary clearance. AS-NS reduced infectious virus in cell monolayers and demonstrated a benign cytotoxicity profile. In human nasal epithelium ex vivo, AS-NS had no impact on mucociliary function (cilia beating nor mucociliary clearance). Carr-NS, HPMC-NS, NO-NS and PI-NS demonstrated limited antiviral effects, while HPMC-NS caused inhibition of mucociliary function. Astodrimer sodium nasal spray demonstrates an acceptable nonclinical efficacy and safety profile as a barrier nasal spray against respiratory viral infection in the nasal cavity.

## Introduction

Since the height of the COVID-19 pandemic, the severity of the condition has lessened, indicated by a decline in mortality rates as well as a reduction in the proportion of patients hospitalized due to COVID-19 and that require intensive medical intervention. This shift in COVID-19 morbidity and mortality patterns can be attributed to the widespread access to highly effective COVID-19 vaccines, a high level of immunity induced by prior infection within the population, availability of outpatient treatment options and changes in the SARS-CoV-2 virus itself^1^. Despite these improvements in managing the spread and impact of COVID-19, it remains a severe condition for many, particularly the elderly, unvaccinated or immunocompromised individuals, and those with respiratory comorbidities. Therefore, the search for effective strategies to complement other preventive and therapeutic options and lessen the incidence and clinical severity of the disease as well as other respiratory infections, including those that may emerge as potential pandemic viruses in future, is ongoing.

Respiratory viruses primarily transmit when an infected individual expels droplets laden with the virus a short distance through the air. These droplets can either directly contact the nasal or oral mucous membranes of a susceptible individual or be indirectly transferred through contact with contaminated surfaces^2–4^. The first line of defence against respiratory viral infections includes intrinsic mechanisms such as mucus production and the innate immune system's detection and response capabilities. The application of a nasal spray, designed to provide a temporary barrier, could enhance these innate defences, potentially curtailing or even averting viral invasion. This intervention might thereby reduce or prevent viral infections and the resultant clinical sequelae.

Astodrimer sodium is a large (3–4 nm, ~ 16.5 kDa), negatively charged, highly-branched dendrimer^5^ with known antiviral and virucidal activity against a broad spectrum of viruses^6–10^. Astodrimer sodium has demonstrated antiviral and virucidal activity against SARS-CoV-2 in vitro^11^, including the Alpha, Beta, Gamma, Delta^12^ and Omicron variants. Astodrimer sodium (1% w/w) in a mucoadhesive nasal spray formulation significantly reduced viral genome copies (> 99.9%) and infectious virus (> 90%) in the lung and trachea compared to saline treatment in K18 hACE2 mice intranasally infected with SARS-CoV-2^13^.

Mechanism of action studies show that astodrimer sodium acts by binding to the highly positively charged regions of the SARS-CoV-2 spike protein to block interaction of the virus with cell-surface heparan sulfate proteoglycans (HSPGs), which precedes the binding of the virus to the angiotensin converting enzyme (ACE) 2 receptor^12^. The interaction of negatively charged astodrimer with the virus spike protein is due to a multivalent electrostatic interaction that is strong and irreversible. Many viruses, including SARS-CoV-2, utilize negatively charged HSPGs protruding from the cell membrane to bind and guide the virus to its specific cellular receptor^14–18^. Astodrimer sodium mimics HSPGs and interferes with these early virus entry steps to block infection.

SARS-CoV-2 receptors have been shown to be highly expressed in nasal epithelial cells^19^, such that intranasally administered therapeutic modalities could be effective in helping to prevent acquisition and spread of viral infection in situations where there is increased risk of exposure. Intranasal formulations must preserve the normal functioning of the nasal system, particularly the mucociliary clearance function. This key nonspecific defence mechanism of the respiratory tract consists of epithelial cilia and mucus secreted by goblet cells. Under normal circumstances, the cilia rhythmically and unidirectionally move mucus—capturing inhaled particles and irritants such as dust, bacteria, viruses, and pollutants—towards drainage sites for removal^20^. As such, any intranasal formulation that negatively impacts ciliary activity or mucociliary clearance risks compromising the innate nasal defence mechanisms^21^.

In this study, the ability of astodrimer sodium nasal spray to block infection by the Delta variant of SARS-CoV-2, recognized as the most concerning in terms of illness severity, was assessed using an in vitro model that is intended to simulate clinical conditions. In addition, the potential effects of astodrimer sodium nasal spray on nasal epithelial integrity and mucociliary clearance function were assessed ex vivo in fully differentiated human nasal epithelial cells. The effects of astodrimer sodium nasal spray were compared with other available nasal spray products. The studies demonstrated the potent ability of astodrimer sodium nasal spray to form a barrier to viral infection, while preserving normal epithelial integrity and mucociliary clearance function.

## Methods

### Nasal spray formulations

The primary nasal spray formulation assessed in this study was astodrimer sodium nasal spray (AS-NS; Viraleze™, Starpharma, Batch Nos. 18482 and 21554). This formulation contains a 10 mg/mL (1%) aqueous solution of astodrimer sodium as the antiviral constituent and carbomer homopolymer type B for mucoadhesive properties. Additional ingredients include glycerol, propylene glycol, methyl p-hydroxybenzoate, propyl p-hydroxybenzoate, and disodium edetate (EDTA).

The following nasal spray formulations containing a diverse range of functional ingredients were also assessed in the study as comparators to astodrimer sodium:

Hydroxypropyl methylcellulose nasal spray (HPMC-NS; Vicks® First Defence, Procter & Gamble, Batch Nos. 1033028830 and 1307028830) is formulated as a low pH nasal spray (pH 3.5) composed of aqua, HPMC (1%), succinic acid (1%), disodium succinate (0.44%), dl-pyrrolidonecarboxylic acid (0.35%), phenethyl alcohol, zinc EDTA, zinc acetate, polysorbate 80, menthol, camphor, sodium saccharin, and eucalyptol.

Carrageenan nasal spray (Carr-NS; mundicare® Cold Defence, Mundipharma, Batch Nos. 9348 and 9305) is a saline solution containing Carragelose™ (iota-carrageenan) at a concentration of 1.2 mg/mL (0.12%).

Nitric oxide nasal spray (NO-NS; Enovid™, SaNOtize, Batch No. 21-987B) is composed of sodium chloride, citric acid, HPMC, sodium nitrite, and benzalkonium chloride.

Povidone iodine nasal spray (PI-NS; CofixRX™ Nasal Solution, CofixRx, Batch No. B8:05/23 JJ) is composed of purified water, povidone-iodine (1.25%), xylitol, vitamin D3, polysorbate, gellan gum, natural carrageenan, citric acid, sodium citrate, glycerin, disodium phosphate, and sodium hydroxide.

### Antiviral barrier function and cell viability

#### SARS-CoV-2 and cells

The SARS-CoV-2 strain, hCoV-19/USA/PHC658/2021 (Lineage B.1.617.2; Delta Variant), was obtained from BEI Resources (NR-55611, Manassas, VA, USA). Virus stock was generated from the supernatant of virus-infected Vero E6 cells (CRL-1586™, ATCC, Washington, DC, USA) maintained in Modified Eagle’s Medium (MEM) supplemented with 10% (v/v) heat-inactivated fetal bovine serum (FBS). Infectious virus titre was determined by plaque assay in Vero E6 cells. SARS-CoV-2 Delta virus stock was used to infect cells in the antiviral assay.

Human lung epithelial Calu-3 cells (HTB-55, ATCC, Washington DC, USA) and Vero E6 cells were used in the antiviral and cell viability assays, respectively. All cells were generated from less than 10 passages. Cells were seeded into 12-well plates at a density of approximately 2.5 × 10^5^ cells per well. Each well contained 1 mL of MEM supplemented with 10% (v/v) heat-inactivated FBS and 1% (w/v) L-glutamine. The cell monolayers were incubated overnight at 37 °C in a 5% CO_2_ environment. All assays were performed on cell monolayers with approximately 80% confluency.

#### Antiviral barrier function

The antiviral barrier function of AS-NS was evaluated in comparison with HPMC-NS, Carr-NS and NO-NS. Phosphate buffered saline (PBS) served as a negative control.

To initiate the assay, the growth media was gently aspirated from the cell cultures. Subsequently, 400 µL of the respective nasal spray or PBS were added to each cell culture to emulate application of the sprays to the nasal epithelium. The cultures were incubated for 5 min on a rocking platform to facilitate the formation of a barrier layer. SARS-CoV-2 was then added, at a multiplicity of infection (MOI) of 0.01, in a maximum volume of 400 µL of MEM. The cell cultures were gently rotated to ensure even coverage of both the cells and the nasal spray layer, and then incubated for 4 h at 37 °C in a 5% CO_2_ environment. This 4 h incubation period aligns with the recommended application of AS-NS every 4 h. Post-incubation, supernatants were recovered to determine the presence of infectious SARS-CoV-2 particles via plaque assay in Vero E6 cells. The antiviral assays were conducted in triplicate.

#### Cell viability

The effects of AS-NS, HPMC-NS, Carr-NS, NO-NS, and PI-NS on cell viability were evaluated in Vero E6 cell monolayers, both quantitatively, by measuring adenosine triphosphate (ATP) production, and qualitatively, through microscopic observation. Each of these cell viability assays was conducted in triplicate.

For the luminescence assay, each nasal spray, in its neat form, underwent serial dilutions to concentrations of 10% (1:10), 3.3% (1:30), 1.1% (1:90), and 0.37% (1:270). Growth media were carefully aspirated from the cell cultures before the addition of 400 µL of each test item dilution. A separate set of three cultures, maintained in the growth media, served as negative controls. The cultures were then incubated for 24 h at 37 °C in a 5% CO_2_ environment. After the incubation, ATP levels, indicative of cell viability, were measured using the CellTiter-Glo® 3D Cell Viability Assay (Promega, Madison, WI, USA, Catalogue No. G9681). This assay involves cell lysis, the use of the released ATP as a substrate for a firefly luciferase reaction, and quantification by luminescence. Cell viability was expressed as a percentage, normalized to the luminescence values obtained from the untreated controls, which were set at 100% cell viability.

For the qualitative assessment of cell viability, both the cell cultures exposed to nasal spray dilutions and the negative controls were examined under 40 × magnification.

### Nasal epithelial integrity and mucociliary function

#### Reconstituted human nasal epithelium

The MucilAir™ system, developed by Epithelix (Geneva, Switzerland), was employed to evaluate the effects of AS-NS on the nasal epithelial integrity and mucociliary function ex vivo. MucilAir is a pseudostratified and ready-to-use, three-dimensional (3D) model of human airway epithelium, comprising primary human epithelial cells freshly isolated from nasal, tracheal or bronchial biopsies. For this study, pooled nasal epithelial cells obtained from 14 healthy adult donors (both male and female) undergoing surgical polypectomy were utilized. Written informed consent was obtained from all these patients. These cells were seeded onto porous inserts (0.33 cm^2^ each, 24-well plates; Transwell™) and cultured at 37 °C and > 95% humidity in a 5% CO_2_ environment, using MucilAir culture medium (EP04MM, Epithelix). Upon reaching confluence, the cultures (hereafter referred to as inserts) were exposed to air on the apical surface while being maintained with medium in the basolateral chamber. This air–liquid interface (ALI) was maintained for 70 days, facilitating appropriate cell differentiation into a mature pseudostratified epithelium consisting of basal cells, ciliated cells, and goblet cells.

Three days before the experiments, inserts were inspected under a conventional inverted microscope to ensure adequate cilia function and the presence of mucus. The inserts selected for the study were washed apically with culture medium to remove any accumulated mucus and cell debris, thereby minimizing the risk of interference with subsequent tests.

#### Study design

AS-NS, HPMC-NS and Carr-NS were applied to MucilAir inserts, with each product being tested at two dose levels: 5 µL and 10 µL. Study endpoints were: cytotoxicity, as determined via lactate dehydrogenase (LDH) assay; tissue integrity, as monitored by trans-epithelial electrical resistance (TEER); and the assessment of effects on cilia, including mucociliary clearance (MCC) and cilia beating frequency (CBF). Two negative controls were employed: untreated inserts and inserts treated with either 5 µL or 10 µL of saline solution. A positive control of 100 µL Triton X-100 was used for the cytotoxicity and tissue integrity assays. For the MCC and CBF assays, a positive control of isoproterenol (50 µM, 1 h exposure) was applied. All the tests were done in triplicate.

The study was conducted in two series. In the first series, the inserts were incubated with the test product for 4 h, which was defined as the treatment phase. In the second series, the treatment phase was extended to a 24 h incubation period. The incubation conditions were 37 °C, 100% humidity, and a 5% CO_2_ environment. At the end of each treatment phase (End of Treatment [EoT], 4 and 24 h, respectively), the inserts were assayed. They were then incubated for an additional 24 h recovery period without test product or control exposure. At the conclusion of the recovery period (End of Recovery [EoR], 28 and 48 h after first product exposure for the first and second series, respectively), the inserts were assayed once more.

Inserts designated for isoproterenol treatment were initially left untreated, with isoproterenol added after 3 h incubation in the first series, restricting its exposure to 1 h. Assays for these isoproterenol-treated controls were performed only at the end of the treatment phase. In contrast, other controls—untreated, saline at 5 µL or 10 µL, and Triton X-100—were applied for the entire duration of the treatment phase across both series. Assays for these controls were conducted at the end of both the treatment and recovery phases.

At each assay timepoint (conclusion of the treatment and recovery phases), procedures were undertaken in the following sequence: measurement of CBF, evaluation of MCC, addition of 200 μL culture media to the apical side and TEER measurement, followed by the removal of apical medium and tissue washing. Lastly, a sample of the culture medium from the basolateral side was taken for LDH analysis.

An overview of the study design is presented in Table 1.

**Table 1:** Overview of the study evaluating the effects of AS-NS, Carr-NS and HPMC-NS on nasal epithelial integrity and mucociliary function.

Schedule		Event
Series 1	Series 2	
0 h	0 h	Start of treatment phase
3 h	NA	Addition of isoproterenol [a]
4 h	24 h	Assay (end of treatment phase) [b]
28 h	48 h	Assay (end of recovery phase) [b]

[a]: Isoproterenol was added only to the Series 1 inserts, which served as positive controls for the MCC and CBF assessments. Note [b]: Assay procedures included measurement of CBF, evaluation of MCC, TEER measurement, and LDH analysis.

#### Tissue integrity (TEER)

Trans-epithelial electrical resistance was measured using an EVOMX volt-ohm-meter (World Precision Instruments UK) at each applicable timepoint. For each MucilAir insert, 200 µL of culture medium was added to the apical surface. The electrodes were placed in the medium on both the apical and basolateral sides of the insert. Subsequently, the resistance values (Ω) were measured at room temperature and later converted to TEER (Ω cm^2^) using Eq. (1), where 100 Ω is the resistance of the membrane and 0.33 cm^2^ is the surface area of the insert.

1 $$TEER \left( {{\Omega }\;{\text{cm}}^{2} } \right) = resistance \left( {\Omega } \right) - 100 \left( {\Omega } \right) \times 0.33 \left( {{\text{cm}}^{2} } \right)$$

Normal values for MucilAir typically fall in the range of 200–600 Ω cm^2^. Higher TEER values (900–1000 Ω cm^2^) may indicate disruptions in ion channels or the destruction of differentiated cells. Conversely, lower TEER values (< 100 Ω cm^2^) are indicative of disruption in epithelial integrity via compromised cellular junctions or the presence of holes in the epithelium. In this study, a positive control treated with 100 µL 10% Triton X-100 was included. Given its cytotoxic properties, Triton was expected to cause a significant disruption in epithelial integrity and, consequently, a decrease in TEER below 100 Ω cm^2^.

Before commencing the study, TEER was measured to ensure that all the selected inserts met the internal quality control standards (> 200 Ω cm^2^).

#### Cytotoxicity (LDH release)

At each applicable timepoint, 50 µL of culture medium was collected and assayed for LDH activity using the Cytotoxicity LDH Assay Kit-WST (cat. no. CK12-20, Dojindo, Rockville, MD, USA) according to the manufacturer's instructions. The high control value was obtained by treating MucilAir inserts apically with 100 µL 10% Triton X-100. The low control value was obtained from untreated inserts, exhibiting a basal LDH release of ≤ 5%, attributable to physiological cell turnover in MucilAir. The released LDH was quantified by measuring the absorbance of each sample at 490 nm using a microplate reader. Cytotoxicity was expressed as a percentage normalized to the high and low controls using Eq. (2), where A represents absorbance.

2 $$Cytotoxicity \left( {\text{\% }} \right) = \frac{{A_{sample} - A_{low} }}{{A_{high} - A_{low} }} \times 100$$

#### Cilia beating frequency (CBF)

The CBF on the apical surface of MucilAir inserts was measured at room temperature with a setup composed of a Sony XCD V60 camera, an Olympus BX51 microscope and a camera-specific software. A total of 256 movies were captured at a high frequency rate of 125 frames per second. The recorded movies were subsequently analysed using the proprietary software Cilia-X (Epithelix), which provided calculated values for CBF expressed in Hz. Normal CBF values for MucilAir typically range from 4 to 8 Hz. For this study, a positive control exposed to 50 µM isoproterenol for 1 h was included. Known for its stimulatory effect, isoproterenol was expected to increase CBF.

Before commencing the study, CBF was measured to ensure that all the selected inserts met the internal quality control standards (> 5 Hz).

#### Mucociliary clearance (MCC)

Mucociliary clearance on the surface of MucilAir inserts was monitored at room temperature using a Sony XCD-U100CR camera, which was connected to an Axiovert 200 M microscope (Zeiss) with a 5 × objective. Polystyrene microbeads of 30 μm diameter (Cat. No. 84135, Sigma-Aldrich, St Louis, MO, USA) were added to the apical surface of the MucilAir inserts. The movement of these microbeads was tracked via video at a rate of 4 frames per second, with 60 images in total being captured. For each insert, three such videos were recorded. Using ImageProPlus 6.0 software (Bioimager, Richmond Hill, ON, Canada), the average velocity of the microbeads' movement (μm/s) was then calculated.

In this study, a positive control was included that had been exposed to 50 µM isoproterenol for 1 h. Known for its stimulatory effect on CBF, isoproterenol was expected to increase MCC.

### Statistical analysis

GraphPad Prism 6 software (San Diego, CA, USA) was used for data handling and graphing. Data were expressed as mean ± standard error of mean (SEM). As and when applicable, differences between three or more groups were tested by one- or two-way analysis of variance (ANOVA) with Dunnett’s or Sidak’s multiple comparison (GraphPad Prism 6). Differences between two groups were tested by Student’s t test. Significance was established at the two-sided significance level of *p* < 0.05.

## Results

### Antiviral barrier function and cell viability

#### Antiviral barrier function

**Fig. 1:**
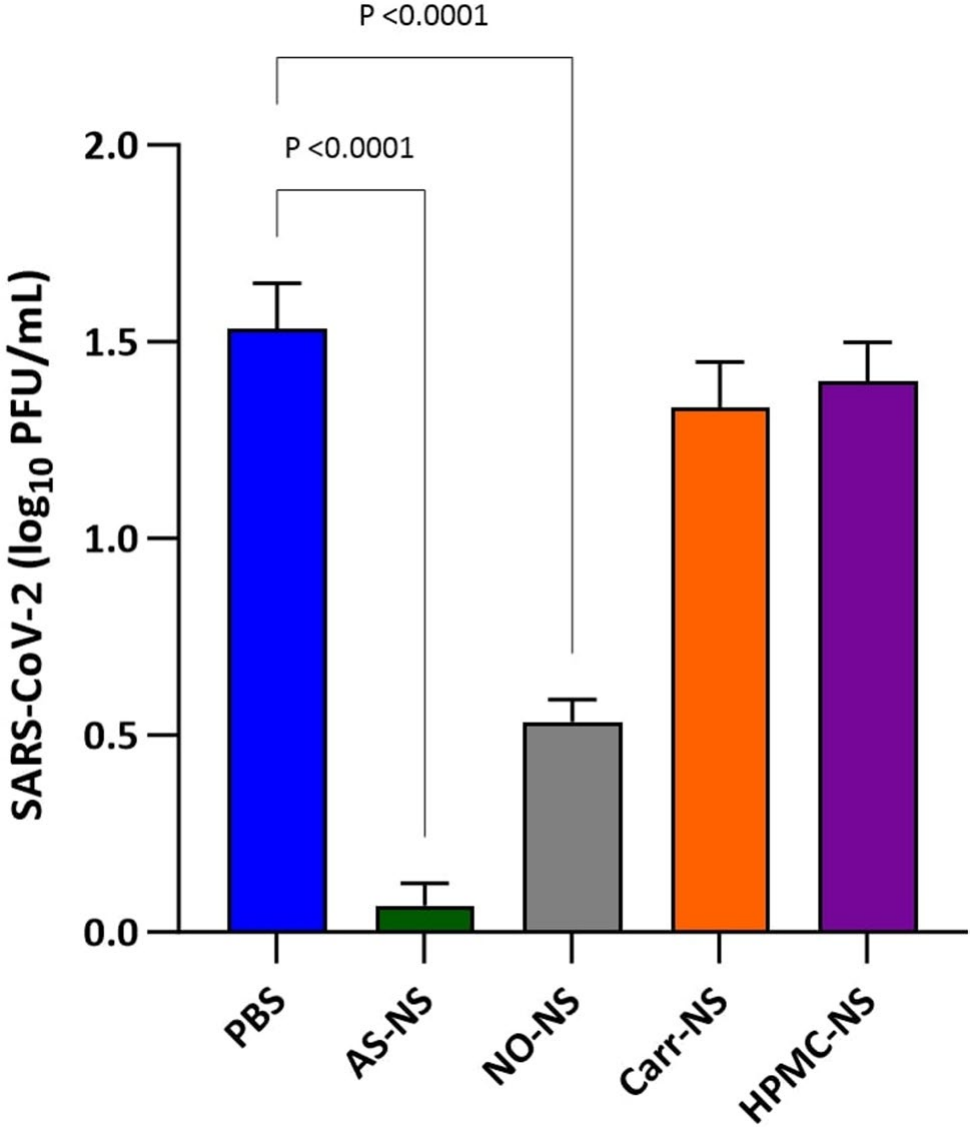
Concentration of infectious SARS-CoV-2 virus, expressed as log_10_ PFU/mL, in supernatants from Calu-3 cell cultures following a 4 h incubation with 400 µL of PBS (negative control), AS-NS, NO-NS, Carr-NS, or HPMC-NS.

AS-NS reduced SARS-CoV-2 viral infectivity/infectious virus by 96.6% compared with PBS-treated negative controls following the 4 h incubation period in Calu-3 cell monolayers (0.07 log_10_ SARS-CoV-2 plaque forming units per mL (PFU/mL) vs 1.53 log_10_ PFU/mL, respectively), and this reduction was statistically significant (*p* < 0.0001). The concentration of infectious SARS-CoV-2 in cells exposed to NO-NS (0.53 log_10_ PFU/mL) was also lower than in PBS-treated cultures (*p* < 0.0001), translating to a 90% reduction in viral load. SARS-CoV-2 infectious virus was not significantly reduced in Carr-NS- and HPMC-NS-treated cultures (1.33 and 1.40 log_10_ PFU/mL, respectively) compared with PBS (Fig. 1).

#### Cell viability

**Fig. 2:**
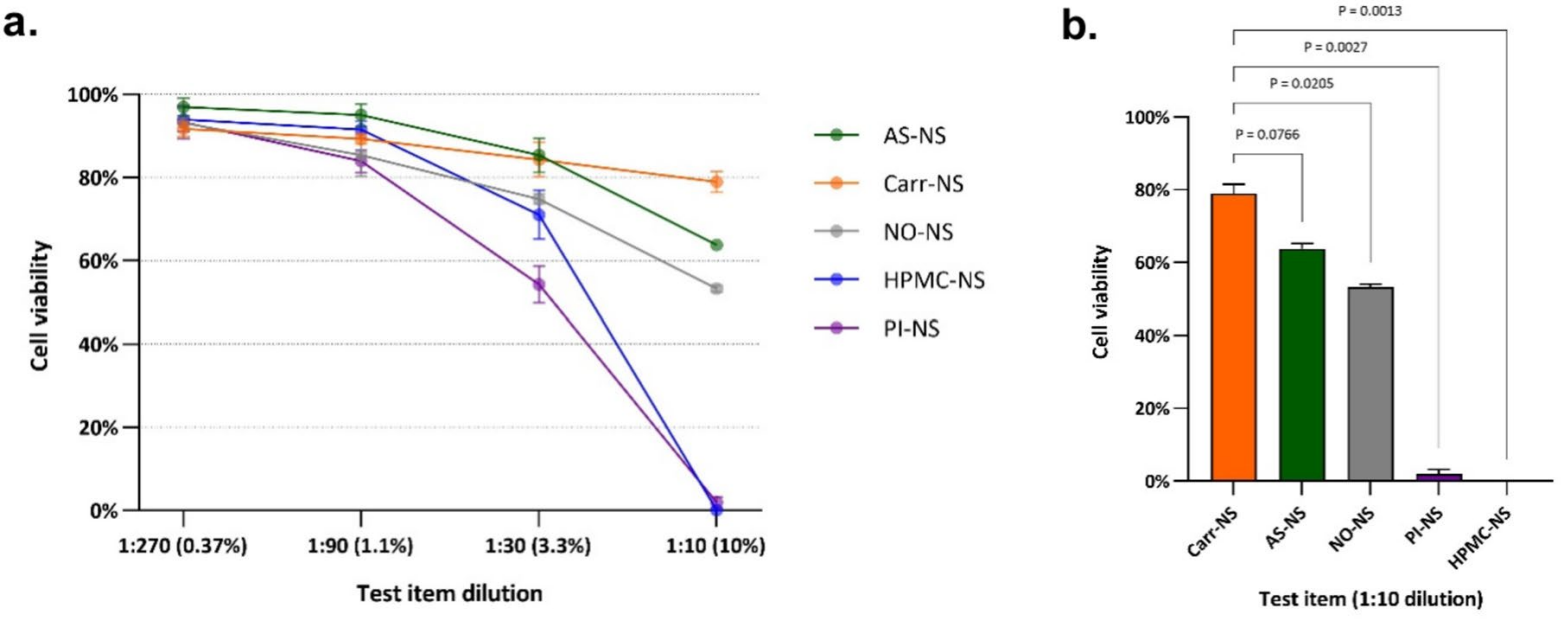
Effects of nasal sprays on cell viability, as determined by ATP-related luminescence, following 24 h exposure of Vero E6 cell monolayers to serial dilutions of AS-NS, Carr-NS, NO-NS, HPMC-NS, and PI-NS (**a**); or to each nasal spray diluted to 10% (**b**). Values are expressed as a percentage of the luminescence observed in untreated controls, corresponding to 100% cell viability.

Cell viability remained near 100%—equivalent to the untreated controls—for AS-NS and all other nasal sprays at dilutions of 0.37% (1:270) and 1.1% (1:90). Notable differences between the test items emerged at the 3.3% (1:30) and 10% (1:10) dilutions. At these dilutions, AS-NS or Carr-NS showed the highest percentage of viable cells, followed by NO-NS. Cell viability was severely reduced by HPMC-NS and PI-NS at 10% (1:10) dilution (Fig. 2a). The differences between Carr-NS and NO-NS, PI-NS and HPMC-NS were statistically significant (Fig. 2b).

**Fig. 3:**
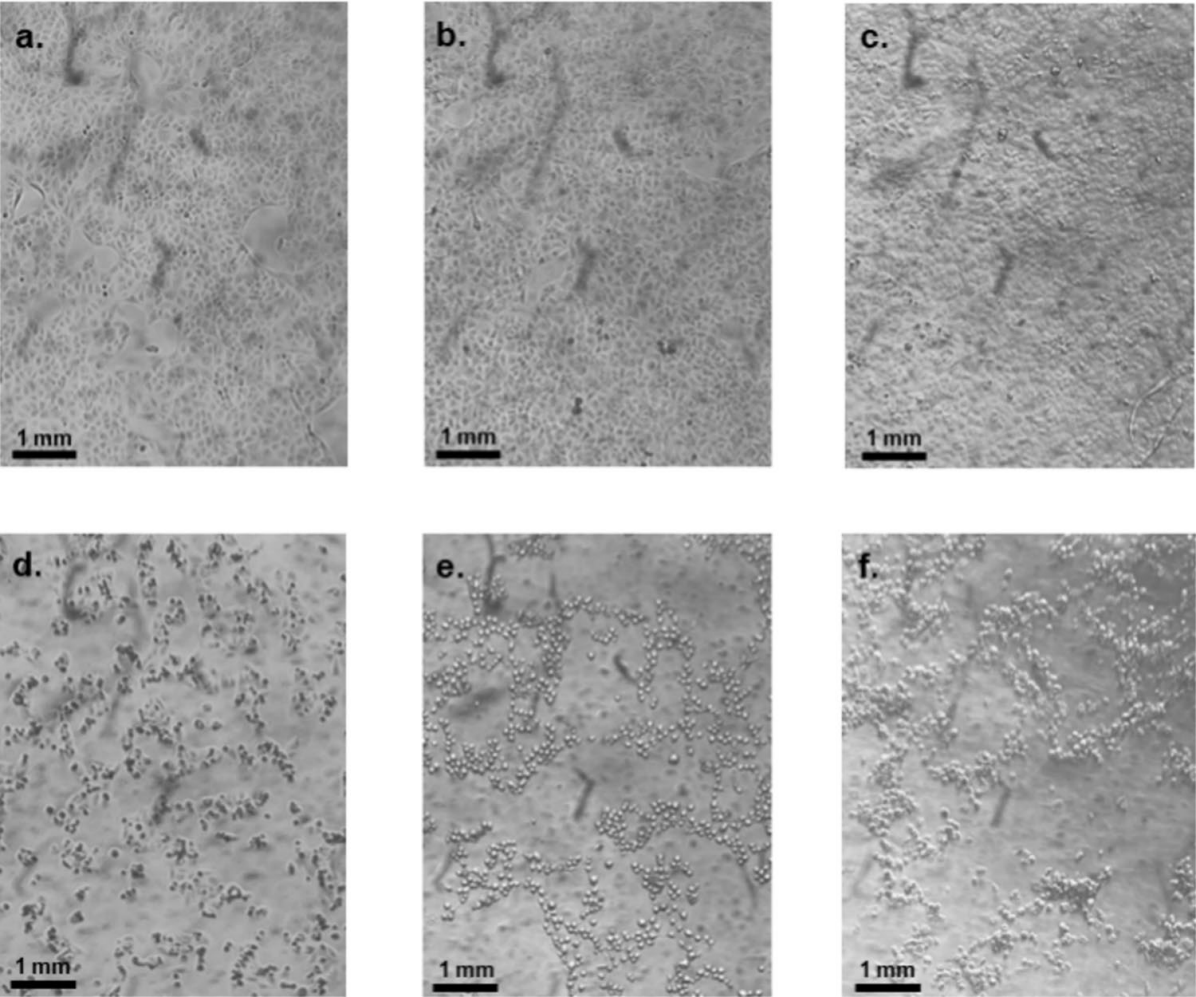
Microphotographs (× 40 magnification) of Vero E6 cell monolayers: untreated (**a**), or after a 24 h exposure to 10% AS-NS (**b**), 10% Carr-NS (**c**), 10% NO-NS (**d**), 10% HPMC-NS (**e**), and 10% PI-NS (**f**). Untreated cells (control), and cells treated with AS-NS or Carr-NS display an intact cell monolayer and appear healthy with no apoptotic or necrotic bodies, while cells treated with NO-NS, HPMC-NS and PI-NS exhibit a rounded shape, reduced cell count, and a disrupted cell monolayer.

The qualitative assessment of cell viability by microscopic observation at × 40 magnification mirrored the quantitative ATP-based results. Untreated Vero E6 cultures, as well as those treated with 10% Carr-NS or 10% AS-NS showed healthy confluent cell monolayers (Fig. 3a–c). In contrast, cultures exposed to 10% NO-NS, 10% HPMC-NS or 10% PI-NS showed severe disruption in the cell monolayer structure (Fig. 3d–f).

### Nasal epithelial integrity and mucociliary function

#### Tissue integrity (TEER)

**Fig. 4:**
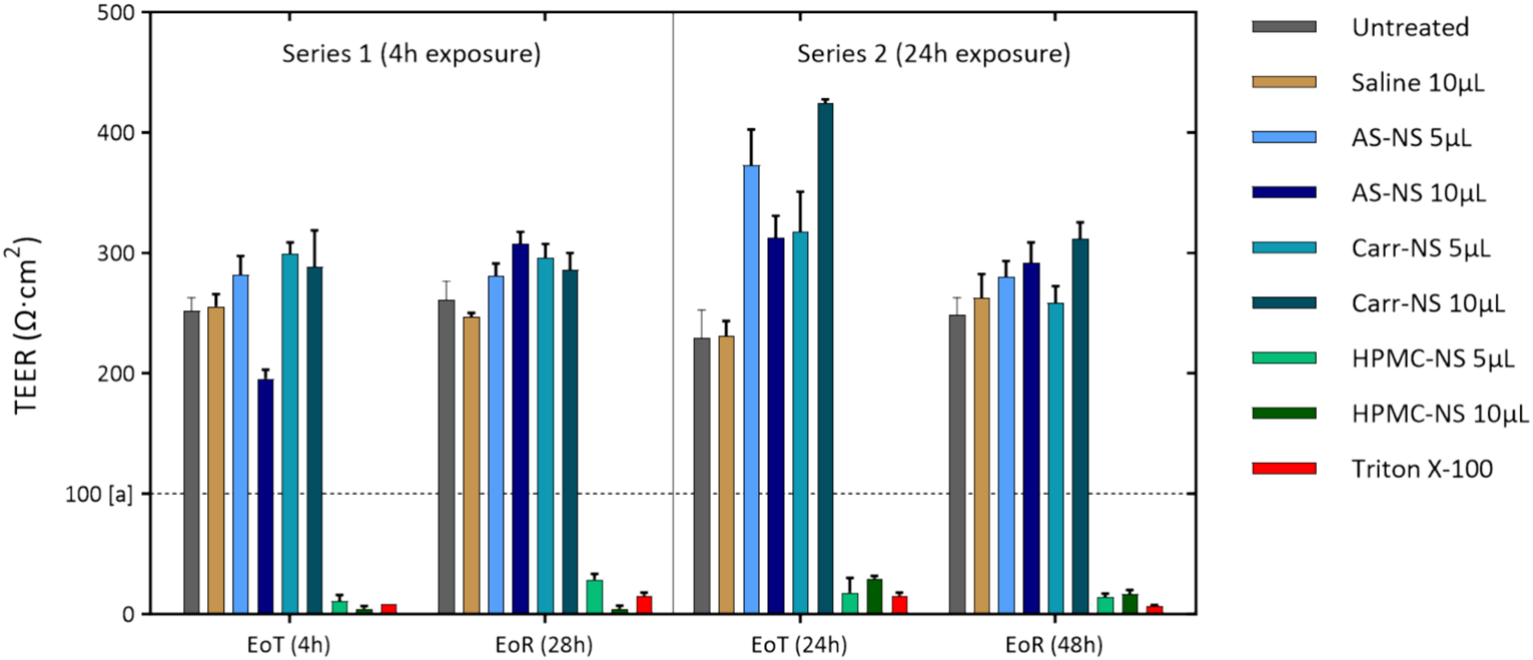
Trans-epithelial electrical resistance (TEER) (indicative of tissue integrity) measured at the end of treatment (EoT) and end of recovery (EoR) in inserts exposed to test items for either 4 or 24 h. Triton X-100 is a positive control, known to induce cytotoxicity mediated epithelial disruption. The dashed line at [a] indicates the critical threshold (100 Ω cm^2^), below which the inserts are considered to display compromised epithelium.

Exposure to 5 or 10 µL of either AS-NS or Carr-NS for 4 h did not compromise the integrity of the nasal epithelium at EoT or at EoR timepoints. The TEER values remained within the normal range well above the critical threshold of 100 Ω cm^2^, and similar to TEER values from untreated controls and saline-treated inserts (Fig. 4).

However, inserts treated with 5 µL HPMC-NS showed TEER values consistent with severe disruption of the nasal epithelial integrity (11.1 ± 2.8 and 28.9 ± 2.6 Ω cm^2^ at EoT and EoR, respectively). Similarly, the TEER values for inserts exposed to 10 µL HPMC-NS were 4.8 ± 1.2 and 5.0 ± 1.2 Ω cm^2^, respectively (Fig. 4).

Both untreated controls and inserts treated with saline solution retained TEER values within the normal range, and inserts exposed to the cytotoxic Triton X-100 exhibited marked epithelial disruption, as evidenced by TEER values below 10 and 20 Ω cm^2^ at EoT and EoR, respectively (Fig. 4).

AS-NS and Carr-NS also showed no evidence of epithelial disruption following an exposure period of 24 h, with TEER results similar to the 4 h exposure series. In contrast, exposure to 5 and 10 µL HPMC-NS for 24 h also resulted in severe epithelial disruption at both EoT and EoR. Untreated controls and inserts exposed to either 5 µL or 10 µL of saline solution for 24 h were unaffected (Fig. 4).

#### Cytotoxicity (LDH release)

No cytotoxicity was observed at any time point for either dosage or exposure time of AS-NS and Carr-NS. In contrast, inserts treated with 5 µL and 10 µL HPMC-NS exhibited significant cytotoxicity at EoT, with some indication of recovery at EoR for the 4 h exposure but worsening of cytotoxicity at EoR for the 24 h exposure (Table 2).

**Table 2:** Observed cytotoxicity at end of treatment (EoR) and end of recovery (EoR) following 4 or 24 h exposure to test items (Series 1 and 2, respectively).

Test item	Cytotoxicity			
	Series 1 (4 h exposure)		Series 2 (24 h exposure)	
	EoT (%)	EoR (%)	EoT (%)	EoR (%)
Saline 5 μL	0.0	0.7	0.0	1.0
Saline 10 μL	0.0	0.0	0.3	0.3
AS-NS 5 μL	0.0	1.0	0.3	3.1
AS-NS 10 μL	0.3	1.4	0.3	2.7
HPMC-NS 5 μL	10.6	4.7	12.4	19.8
HPMC-NS 10 μL	14.3	4.7	10.0	25.6
Carr-NS 5 μL	0.0	1.0	0.0	0.7
Carr-NS 10 μL	0.0	0.0	0.0	1.4

Data are presented as the percentage of cell death, normalized to the range defined between the cytotoxicity reading obtained with untreated inserts (considered to be 0%) and the cytotoxicity reading obtained from inserts treated with the cytotoxic Triton X-100 (considered to be 100%).

Marginal increases in cytotoxicity for saline and other products were deemed biologically irrelevant and were attributed to background variability.

#### Cilia beating frequency (CBF)

**Fig. 5:**
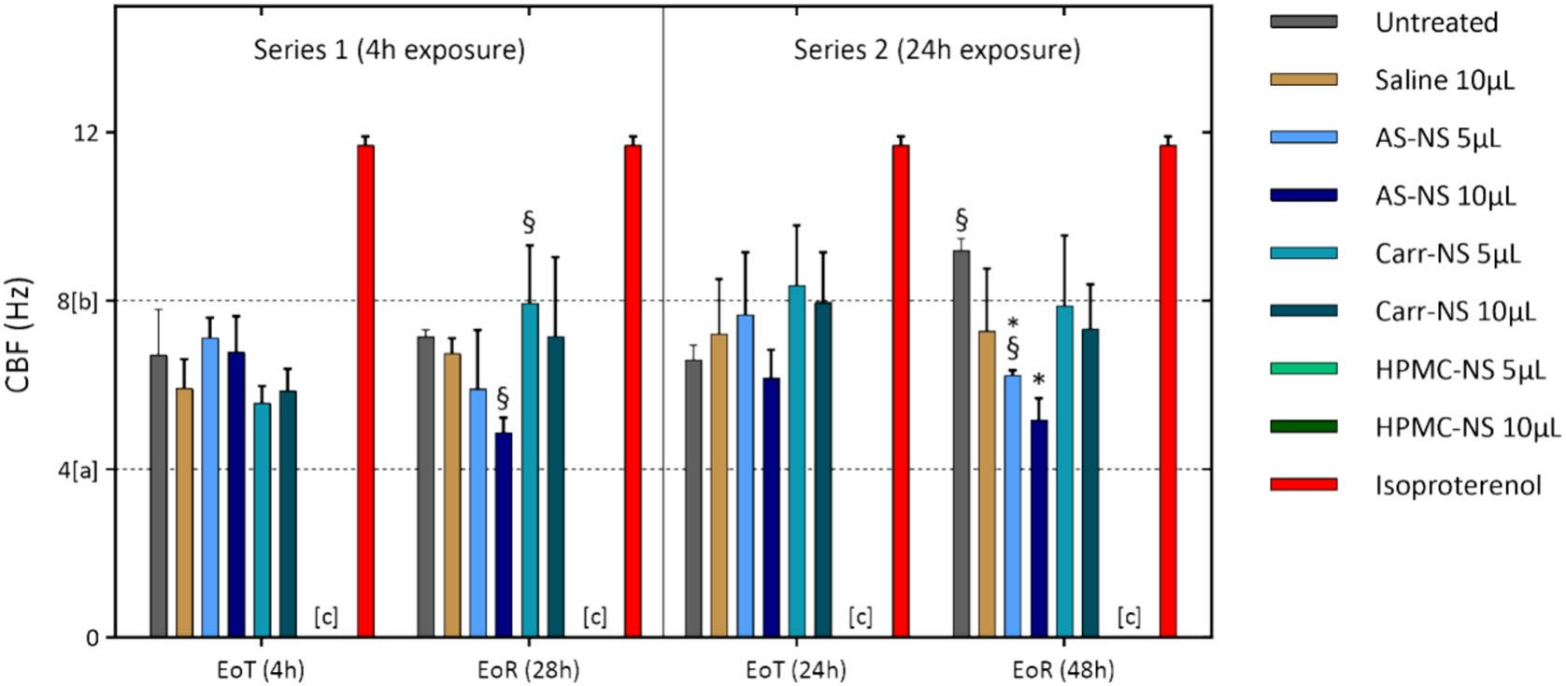
CBF at EoT and EoR in inserts exposed to test items for either 4 or 24 h. Isoproterenol serves as a positive control, inducing enhanced ciliary beating. The dashed lines at [a] (4 Hz) and [b] (8 Hz) indicate the standard range of CBF for the MucilAir™ system. Note [c]: Bars representing CBF for HPMC-NS at either 5 or 10 µL are not plotted due to the total loss of ciliary beating after exposure to this test item, reflecting a CBF value of 0 Hz. This difference was statistically significant (*p* < 0.0001) when compared with untreated controls/saline. *p < 0.05 relative to untreated inserts at the corresponding timepoint (EoT or EoR). §*p* < 0.05 relative to the same treatment at EoT.

AS-NS and Carr-NS (5 μL or 10 μL) had no impact on CBF at EoT following a 4 h exposure, with CBF remaining within the normal range (4–8 Hz) for the MucilAir system (Fig. 5).

In contrast, all inserts treated with HPMC-NS exhibited complete absence of ciliary motion at both EoT and EoR. As expected, inserts treated with the positive control, isoproterenol, showed an increased CBF vs untreated control/saline (*p* < 0.001) (Fig. 5).

AS-NS-treated inserts showed slightly lower CBF values at EoR compared to EoT, with this decrease being statistically significant for inserts treated with 10 μL AS-NS (6.8 vs. 4.9 Hz; *p* < 0.01). Despite this, CBF remained within normal limits and did not lead to decreased mucociliary clearance relative to untreated or saline-treated controls. Conversely, Carr-NS-treated inserts showed elevated CBF at EoR compared to EoT, with this increase being statistically significant for inserts treated with 5 μL Carr-NS (5.6 vs. 8.0 Hz; *p* < 0.001) (Fig. 5).

Results following 24 h exposure to test items concurred with those from the 4 h exposure series. CBF values remained within or slightly above the normal range for untreated inserts and those treated with either volume of saline solution, AS-NS, or Carr-NS at either EoT or EoR. Exposure to HPMC-NS for 24 h also resulted in a complete loss of ciliary beating (Fig. 5).

Consistent with the other observations, AS-NS-treated inserts showed slightly lower CBF at EoR compared to EoT, with a significant difference in the 5 μL AS-NS group (7.7 vs. 6.2; *p* < 0.01). Additionally, untreated inserts displayed a statistically significant higher CBF at EoR compared to EoT (6.6 vs. 9.2; *p* < 0.001). Comparing the unusually high CBF at EoR in untreated inserts with the relatively low values recorded in AS-NS-treated inserts rendered the latter significantly lower (9.2 Hz vs. 6.2 for 5 μL, *P* = 0.01; and vs. 5.2 for 10 μL, *p* < 0.001). This finding was considered irrelevant as the CBF for AS-NS-treated inserts remained within the normal range (Fig. 5).

#### Mucociliary clearance (MCC)

**Fig. 6:**
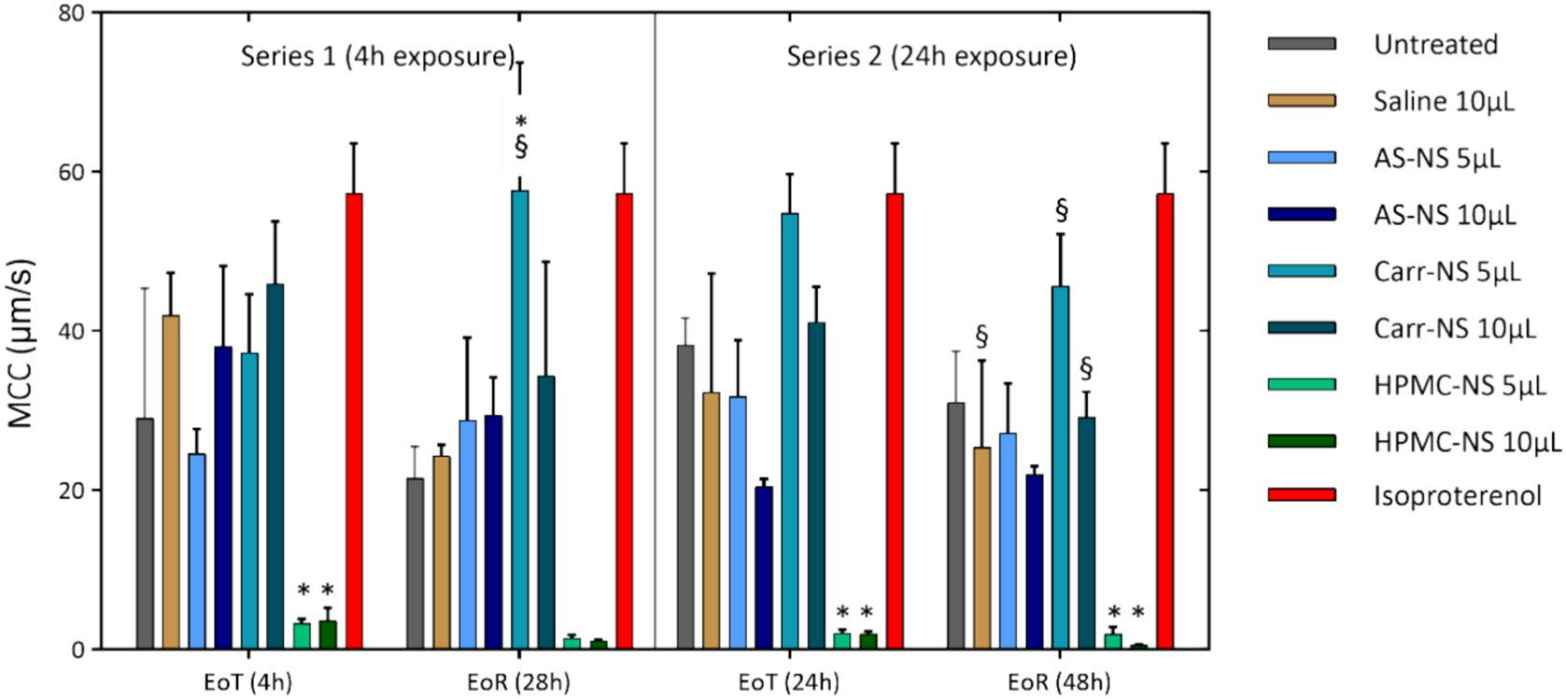
MCC at EoT and EoR in inserts exposed to test items for either 4 or 24 h. Isoproterenol is a positive control, known to enhance MCC through its stimulatory effect on ciliary beating. *p < 0.05 relative to untreated inserts at the corresponding timepoint (EoT or EoR). §*p* < 0.05 relative to the same treatment at EoT.

In the 4 h exposure series, untreated controls showed particle velocities of 29.0 μm/s and 21.5 μm/s at EoT and EoR, respectively. Inserts treated with saline exhibited higher velocities than untreated controls at EoT (44.2 and 42.0 µm/s for 5 and 10 µL, respectively). At EoR, velocities had decreased and were similar to those of untreated controls (23.1 and 24.3 µm/s for 5 and 10 µL) (Fig. 6).

Velocities recorded in AS-NS-treated inserts were similar to those of untreated inserts across all exposure volumes and timepoints, ranging from 24.6 µm/s (5 µL at EoT) to 38.0 µm/s (10 µL at EoT). Carr-NS-treated inserts showed higher velocities than controls, with values of 37.3 and 46.0 µm/s for 5 and 10 µL at EoT, and 57.7 and 34.3 µm/s at EoR. Consistent with the absence of ciliary beating, HPMC-NS-treated inserts exhibited markedly low velocities at both EoT (< 4 µm/s) and EoR (< 2 µm/s), indicative of a loss of the MCC function (Fig. 6).

In the 24 h exposure series, untreated controls and saline-treated inserts showed comparable velocities at both EoT and EoR, ranging from 25.3 µm/s (10 µL saline at EoR) to 38.2 µm/s (untreated control at EoT). While 5 µL AS-NS-treated inserts recorded velocities consistent with untreated controls and saline solution-treated inserts, 10 µL AS-NS-treated inserts presented slightly lower velocities (20.4 and 21.9 µm/s at EoT and EoR, respectively), although the differences were not statistically significant. In agreement with the 4 h exposure results, Carr-NS-treated inserts exhibited higher velocities than controls, while HPMC-NS-treated inserts showed a loss of MCC, evidenced by particle velocities below 2 µm/s (Fig. 6).

## Discussion

Astodrimer sodium, a novel, highly branched, anionic, polyvalent molecule, has been formulated in a mucoadhesive nasal spray to act as a barrier to prevent respiratory viruses from reaching host target cells. In these studies, designed to closely resemble the conditions of intended clinical use, astodrimer sodium has been shown to act as a functional antiviral barrier while preserving the viability of human airway epithelial cell monolayers in vitro and maintaining the mucociliary function and integrity of fully differentiated human nasal epithelium ex vivo.

The antiviral barrier function study in cell monolayers was designed to emulate clinical conditions in which AS-NS forms a mucoadhesive barrier on the nasal epithelium, physically blocking the binding of the positively charged SARS-CoV-2 spike protein to negatively charged regions of the host cell and its receptor-complex. Human Calu-3 lung epithelial cells, which produce mucous proteins important for mucoadhesive barrier formation, were used to closely mimic the biological situation. These cell cultures were maintained in a minimal volume of media to enhance the barrier formation. The SARS-CoV-2 Delta variant was chosen because it is highly transmissible and replicates well in Calu-3 cells, as they are susceptible to infection via the serine-protease mediated entry pathway. After nasal spray application, cultures underwent a 4 h incubation with the virus. This exposure time aligns with the recommended application of AS-NS every 4 h. The selected incubation period is considerably shorter than the estimated ~ 10 h eclipse period for SARS-CoV-2^22^. Consequently, the infectious viral particles quantified by standard plaque assay are not attributable to progeny virus but to the viral inoculum to which the cultures were exposed.

AS-NS exhibited potent antiviral barrier effects, as evidenced by a 96.6% reduction (1.5 logs) of SARS-CoV-2 infectious virus. A significant reduction in infectious virus was also observed for NO-NS, with a decrease of 90.0% (1 log). Conversely, minimal antiviral activity was observed after exposure to Carr-NS and HPMC-NS. In previous research involving SARS-CoV-2-infected Vero E6 and Calu-3 cells treated with 10 mg/mL astodrimer sodium in aqueous solution (the same concentration as in AS-NS), reductions of infectious particles by 5 (99.999%) and 3 (99.9%) logs were reported, respectively^11^. The smaller reductions noted in the current study are not indicative of a lesser efficacy of AS-NS but rather stem from differences in the experimental design. The current study aimed to mirror clinical conditions, leading to the selection of a lower virus inoculum and a shorter incubation period compared with previous studies. Consequently, a lower viral concentration was present in collected supernatants, limiting the observable extent of the antiviral effects of AS-NS. The formation of a nasal spray barrier in vivo is likely to be less uniform than achieved in the current study. Nevertheless, the results of the current study are consistent with nonclinical studies in mice showing that AS-NS reduces SARS-CoV-2 load in lung, trachea, brain and liver in vivo^13^, and a clinical study of efficacy of AS-NS in patients with COVID-19 has been conducted with results yet to be published.

In the cell viability assay conducted on Vero E6 cell monolayers, AS-NS and Carr-NS were relatively benign among the tested nasal sprays, with minimal effects on cell viability. NO-NS resulted in a lower cell viability, while extremely low cell viabilities were observed post-exposure to PI-NS (2%) and HPMC-NS (nearly zero). Under × 40 magnification, monolayers exposed to either Carr-NS or AS-NS were found to maintain their confluent structure. In contrast, substantial disruption was evident in cultures exposed to NO-NS, PI-NS, or HPMC-NS.

The series of ex vivo assays to characterize the potential adverse effects of AS-NS on nasal epithelia integrity and mucociliary clearance function were also designed to mimic the intended clinical scenario. The MucilAir cell cultures (or inserts) employed in these evaluations consisted of fully differentiated and functional human pseudostratified nasal epithelium, which included basal cells, ciliated cells, and goblet cells. To simulate intermittent and prolonged nasal delivery, undiluted nasal spray formulations were administered apically at volumes of 5 and 10 μL and exposure durations of 4 and 24 h. Notably, the volume-to-surface ratios utilized in this study mirror those in a typical clinical setting, underscoring their relevance in assessing the safety profiles of the nasal sprays investigated. The average surface area of the adult nasal cavity is 160 cm^2^, with only the anterior third being accessible to nasal sprays^23–25^. Considering that AS-NS is administered via two 100 μL actuations (one for each nostril) up to four times daily, the volume of AS-NS delivered per unit area of the nasal epithelium would be about 15 μL/cm^2^. In this study, the selected 5 μL dose over the 0.33 cm^2^ surface of the MucilAir inserts also yields a volume-to-surface ratio of approximately 15 μL/cm^2^. The 10 μL dose doubles the anticipated clinical exposure.

No clinically significant alterations in nasal epithelia integrity were observed following exposure to AS-NS or Carr-NS with TEER remaining above the critical threshold of 100 Ω cm^2^, and there were no dose- or time-dependent effects. In contrast, exposure to HPMC-NS led to a severe disruption of tissue integrity.

Cytotoxic assessments in MucilAir inserts, measured via LDH release, were consistent with the cell viability results from Vero E6 cell monolayers. AS-NS and Carr-NS were not cytotoxic, whereas significant cytotoxicity was observed for HPMC-NS. After the off-treatment recovery period, a partial recovery was noted in inserts exposed to HPMC-NS for 4 h. In contrast, inserts exposed to HPMC-NS for 24 h not only did not recover but exhibited further deterioration. Such findings align with the epithelial disruption observed post-exposure to HPMC-NS and indicate that, in this model, prolonged exposure to HPMC-NS might hinder natural tissue repair processes.

Following exposure to AS-NS and Carr-NS, epithelial mucociliary function, as determined by cilia beating frequency and mucociliary clearance, remained within the normal range. However, exposure to HPMC-NS resulted in the complete cessation of cilia activity and a dramatic reduction in MCC. Interestingly, though not statistically significant, a heightened MCC was observed following the 4 h exposure to the high dose (10 µL) of saline, AS-NS, or Carr-NS, compared to the low dose (5 µL). This might be attributed to a mucus dilution effect induced by the larger volume, leading to a less viscous mucus that is easier to move. This trend was not evident after the 24 h exposure or at the conclusion of the off-treatment recovery periods, possibly because the inserts had sufficiently adjusted to the added volume. Notably, inserts treated with the 5 µL dose of Carr-NS exhibited elevated MCC values both after the 24 h exposure and at the end of the recovery periods. The reasons underpinning these observations remain to be elucidated.

The results of these studies showing no adverse effects of AS-NS on human nasal epithelium are consistent with clinical data on the nasal spray in humans, which demonstrated that AS-NS is well-tolerated and not systemically absorbed, with only mild local side effects and no systemic adverse events observed following dosing 4 times daily for 14 days^26^. Similarly, clinical data for Carr-NS indicate a well-tolerated safety profile^27^ consistent with the data observed in the human epithelial model in this study. There is limited published clinical data available for HPMC-NS, a product intentionally designed with a low pH to cause a mild sensation of irritation in the nasal cavity, aimed at stimulating the production of nasal secretions and mucus to increase clearance^28^. The adverse effects observed in vitro and in the MucilAir nasal epithelial model in this study may be a consequence of this intended effect.

## Conclusions

These studies indicate that astodrimer sodium nasal spray has the potential to act as a barrier to viral infection while preserving normal mucociliary function when applied as a spray into the nasal cavity. In contrast, other nasal sprays tested showed variable antiviral barrier properties and mixed effects on cell viability and mucociliary function. The combination of antiviral properties and the preservation of normal nasal epithelial mucociliary function makes astodrimer sodium nasal spray a promising option for reducing exposure to infectious respiratory viruses. Such nasal sprays can be employed prophylactically or as post-infection treatment to decrease exposure to viral load in the nasal cavity, potentially preventing, or alleviating the severity and duration of respiratory viral infection symptoms.

## Data Availability

All data are included in the manuscript and supplementary materials.
